# Viscoelasticity Modeling of Dielectric Elastomers by Kelvin Voigt-Generalized Maxwell Model

**DOI:** 10.3390/polym13132203

**Published:** 2021-07-02

**Authors:** TuanDung Nguyen, Jin Li, Lijie Sun, DanhQuang Tran, Fuzhen Xuan

**Affiliations:** School of Mechanical and Power Engineering, East China University of Science and Technology, 130 Meilong Rd., Shanghai 200237, China; tuandungbkth1010@gmail.com (T.N.); y30180352@mail.ecust.edu.cn (L.S.); danhquangtran4788@gmail.com (D.T.); 193811756@gmail.com (F.X.)

**Keywords:** dielectric elastomer, electromechanical coupling, creep, viscoelastic electromechanical modeling, soft robotics

## Abstract

Dielectric elastomers (DEs) are polymer materials consisting of a network of polymer chains connected by covalent cross-links. This type of structural feature allows DEs to generate large displacement outputs owing to the nonlinear electromechanical coupling and time-dependent viscoelastic behavior. The major challenge is to properly actuate the nonlinear soft materials in applications of robotic manipulations. To characterize the complex time-dependent viscoelasticity of the DEs, a nonlinear rheological model is proposed to describe the time-dependent viscoelastic behaviors of DEs by combining the advantages of the Kelvin–Voigt model and the generalized Maxwell model. We adopt a Monte Carlo statistical simulation method as an auxiliary method, to the best knowledge of the author which has never reportedly been used in this field, to improve the quantitative prediction ability of the generalized model. The proposed model can simultaneously describe the DE deformation processes under step voltage and alternating voltage excitation. Comparisons between the numerical simulation results and experimental data demonstrate the effectiveness of the proposed generalized rheological model with a maximum prediction error of 3.762% and root-mean-square prediction error of 9.03%. The results presented herein can provide theoretical guidance for the design of viscoelastic DE actuators and serve as a basis for manipulation control to suppress the viscoelastic creep and increase the speed response of the dielectric elastomer actuators (DEA).

## 1. Introduction

Dielectric elastomers (DEs) are a combination of dielectric electroactive polymers. The DE actuator structure consists of a thin membrane of elastomer sandwiched between two compliant electrodes. When subjected to a voltage across its thickness, such a material expands in area and shrinks in thickness based on the effects of Maxwell stress [1,2]. This behavior can facilitate intriguing muscle-like behavior for the development of soft robots. Dielectric elastomer actuators (DEAs) are a type of soft material actuator that can deform in response to voltage [3,4]. Compared to other smart elastomer materials, DEs exhibit desirable attributes, such as high strain rates (up to 380%), high efficiency (up to 90%), high energy density (3.4 J/g), low modulus, simple structure, and excellent environmental compliance [5,6,7]. Therefore, DEs have artificial muscle properties and are widely used in robotic fields for the development of jellyfish robots [8], hexapod robots [9], annelid robots [10], and wall climbing robots [11], and have been extensively used in many scientific fields for the development of artificial muscles, soft sensors, optical devices, and energy generators.

### 1.1. Background

Although the geometrical structure and working principle of DEs are relatively simple, the material’s deformation behavior is very complex because of the hyperelastic and nonlinear electromechanical coupling characteristics of the material [12]. The dissipative properties of DEs include viscoelasticity, dielectric relaxation, and conductive relaxation. These nonlinear properties enhance the difficulty of dynamic modeling [13,14]. Experimental results have shown that the stress-strain curves of DEs are closely related to tensile rates [15], and the strain under-voltage excitation is closely related to time [16,17]. Additionally, DEs exhibit viscoelasticity, which is a strongly time-dependent behavior. Therefore, Hook’s law for traditional linear-elastic material models cannot be used to describe the electromechanical coupling constitutive relationships of DEs. Therefore, the challenge is how to develop a model that can accurately describe the time-dependent viscoelastic response behavior of dielectric elastomer materials.

### 1.2. Related Work

Many researchers have established viscoelastic models for DEs. These models can be divided into three main groups as follows.

(1) Conventional models are based on the mechanical and physical properties of the DE materials. Wissler and Mazza [18] used a Prony series to establish a linear viscoelastic model. Afterward, linear rheological models were established to describe the time-dependent viscoelastic properties of the materials [19,20]. The development of these models represents the start of the DE material modeling revolution. However, such models may have difficulty describing the complex nonlinear behaviors of DE materials, especially under large loads. A nonlinear viscoelastic model was developed based on Christensen’s theory [21] to improve the accuracy of such models. Lochmatter et al. [22] investigated the viscoelastic properties of a VHB4910 film and used a novel model to describe the time-dependent mechanical behavior of DEs. Although these models have been able to describe the nonlinear behavior of DEs, their simplicity can negatively affect the accuracy of model descriptions.

(2) As a breakthrough in the research history of DEs, by combining the Maxwell and Kelvin models, Hong [23] reported that DEs could be approximately represented by rheological models, including an array of springs and dashpots, which constitute the standard linear solid model. The establishment of this model was a turning point in the DE research. Many studies have attempted to improve the accuracy of model descriptions. A spring combined with a damped series system can form a Maxwell rheological model, and a Kelvin model can be formed in parallel. By combining these two types of models, Zhang [24] proposed the Kelvin-Voigt-Maxwell model to describe the entire process of DE deformation under DC voltage excitation. However, these models are relatively simple and are characterized by a single relaxation time parameter. Therefore, it is difficult to describe the rheological deformation processes of DE systems under complex loading, and such models cannot accurately characterize the viscoelastic time characteristics.

(3) The development of generalized rheological models has improved the precision of viscoelastic behavior evaluation. Generalized models with multiple relaxation times have also been developed to improve the accuracy of viscoelastic effect prediction. Khan et al. [25] established a generalized Maxwell (GM) model for viscoelastic DEs, and the results showed strong agreement with the experimental data. Gu et al. developed a multi-relaxation time rheological model to predict the viscoelastic effects of materials more accurately. However, in that study, only alternating voltage loading was considered, and inertial force was ignored.

### 1.3. Study Contributions

A high-accuracy rheological model is proposed in this study to improve the prediction ability of rheological models under complex loading conditions. We combine the Kelvin-Voigt model and the GM model to develop the KV-GM model. The main contributions of this study are summarized as follows.

(1) A generalized rheological KV-GM model is presented. This model combines the advantages of two classical composite models with multiple relaxation times to describe the complex nonlinear viscoelastic properties of DEs.

(2) Based on the principles of virtual work and non-equilibrium thermodynamics, a constitutive model of viscoelastic DEs under-step voltage excitation was established. Under the excitation of alternating voltage, to improve the physical characteristics of the system, the work done by inertial forces is considered to form a dynamic model of the DE system.

(3) Parameter identification of the generalized rheological model adopts a parameter analysis method combined with Monte Carlo statistical simulation; the latter is used as an auxiliary method. This type of combinatory analysis has never been adopted to obtain a set of optimal model parameters to improve the quantitative prediction ability of the generalized model.

The remainder of this paper is organized as follows. The DE system and the experimental configuration are introduced in Section 2. Section 3 presents the experimental results under different steps and alternating voltage signal loads. In Section 4, we propose the theory of viscoelasticity and establish a constitutive model. The Monte Carlo method and model parameter analysis is applied in Section 5 to obtain quantitative description parameters to improve the model prediction capabilities. Section 6 concludes the paper.

**Figure 1:**
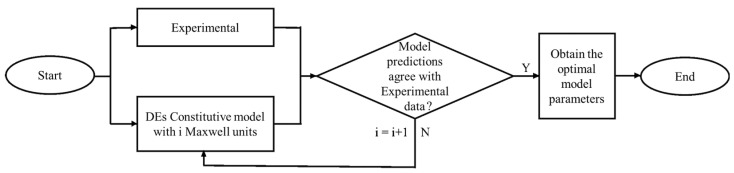
Research Flowchart of dielectric elastomers (DE).

The course of the technology research of this paper is shown in Figure 1.

## 2. Experimental Description

### 2.1. De Membrane Actuator

**Figure 2:**
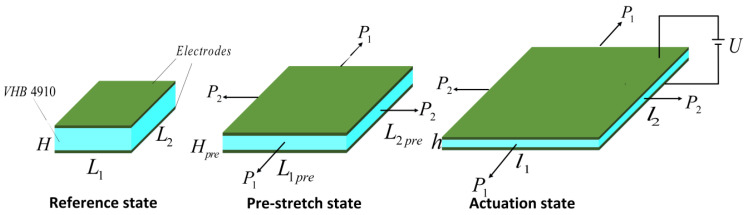
DE membrane in the reference state, pre-stretch state, and actuation state.

In this paper, a square DEA was fabricated as shown in Figure 2, which was able to provide linear actuation with 2 DOF. In the reference state, the length of the DEA was $L_{1}=L_{2}=10\;\mathrm{mm}$ and thickness H was 1 mm. The material used for this actuator was an acrylic elastomer VHB4910 (3M Company, Saint Paul, MN, USA). The film was subjected to uniform biaxial pre-stretching $\lambda_{p1}=\lambda_{p2}=3$ and fixed to a rigid frame. In the pre-stretched state, the length of the DEA was $L_{1pre}=L_{2pre}=30mm$. In order for the VHB4910 material to be incompressible, the thickness of the DEA changed to $H_{pre}=H/\lambda_{p1}\lambda_{p2}$. In the actuated state, subjected to a voltage U through the thickness of the DEA, the electrons flowed from one electrode to each other, and the two electrodes gained the charge +Q and −Q, respectively. Due to the Maxwell stress, the DEA deformed to a new configuration of thickness $H=h/\lambda_{1}\lambda_{2}$ and two dimensions of lengths $l_{1}=L_{1}\lambda_{1}$, $l_{2}=L_{2}\lambda_{2}$, respectively.

### 2.2. Experimental Setup

**Figure 3:**
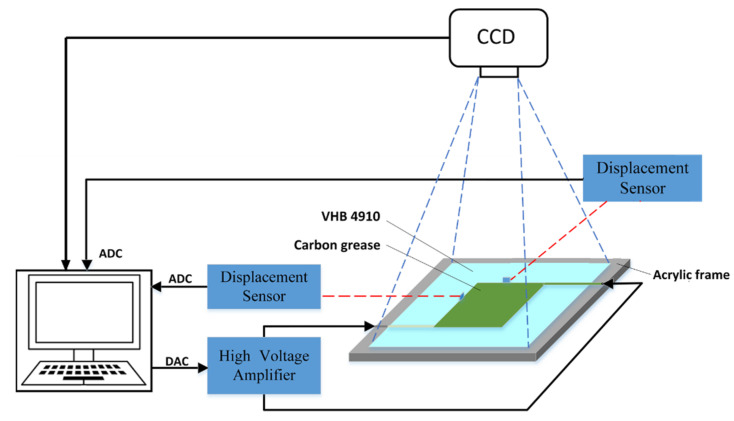
The experimental setup.

Figure 3 presents a block diagram of our experiment setup, which consisted of a dSPACE_DS1103 control board with 16-bit digital-to-analog converters for generating an analog control voltage, which was fed into a high-voltage amplifier (10/40A, Trek, Inc. Waterloo, WI, USA) to amplify the voltage signal with a fixed gain of 4000. A laser sensor (LK_G4000A, Keyence, Osaka, Japan) measured the real-time displacement of the DEA. The laser sensor was connected to a set of 16-bit analog-to-digital converters (ADCs). Output displacement data were recorded and converted into small voltage signals (0 to 1 V) by a control box, and the experimental data were sent to a computer for display by the ADCs. Additionally, MATLAB/Simulink software (Natick, MA, USA) was used to implement the algorithms. This software transmitted control signals to the control board through the control desk interface to conduct voltage signals and accept output displacement signals in real-time. Our experiments were all performed at room temperature, so the influence of temperature variation on the experimental results could be ignored.

To measure the area deformation of the DEA during the excitation process, as shown in Figure 3, the CCD external trigger camera (DS-CFST140M-H4, Do3think, Shenzhen, China), with an accuracy of 1360 × 1024 pixels was used to record the deformation of the DEA in real-time, and BasedCAM software was used to control the characteristics of the output images and adjust the time interval. In this experiment, when taking continuous images with a CCD camera, the minimum time interval for saving two images was set to 1 s. The obtained images were processed by MATLAB Image Processing Toolbox (Natick, MA, USA), after binarization of the image, the pixel points of the driving region could be calculated to obtain the area of the target region. To get the area of the actuator, a square sample of 1 cm × 1 cm was used as a test for the CCD camera. With the same camera setup and image processing method, the ratio between the actual area and the image target area could be obtained.

### 2.3. Experimental Results

**Figure 4:**
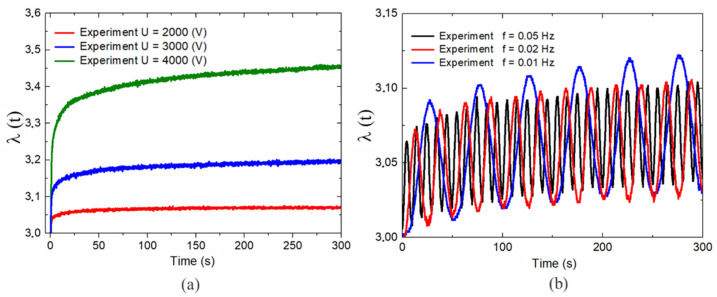
Experimental results: (**a**) step response and (**b**) alternating response with U=2000sin2πft (V).

The experimental platform is shown in Figure 4. In this study, we mainly focused on the square DEA responses under step and alternating voltage excitation. To avoid instability in the actuator, the step and alternating voltages adopted here were less than the critical values of $U_{step}=4\mathrm{kV}$ and $U_{\mathrm{alternating}}=U_{0}\sin(2\pi ft)$, respectively, where $U_{0}=2\mathrm{kV}$ and f=0.01, 0.02, and 0.05 Hz. The experimental results demonstrated that the step response and alternating response were time-dependent deformations under complex load excitation. For simplicity, the response process can be divided into three stages. Stage I was a creep stage with a few hundred seconds of step responses [26] and approximately three periods of alternating responses [27]. Stage II was a relaxation stage that lasted for a long duration. Stage III was the stable stage.

As shown in Figure 4a, the response of the system became more complex as the excitation voltage increased. Additionally, with an increase in the excitation voltage, the relaxation response process of the system became more complicated, and it took longer for the system to reach a stable stage. Figure 4b presents the dynamic responses of the DE system under the excitation of a sinusoidal alternating voltage with varying frequencies. These results demonstrated that the response behavior of the DE system was frequency-dependent. The maximum amplitude of the actuation strain was obtained when f=0.01 Hz and tended to decrease as the frequency increased. This phenomenon is explained by the fact that the frequency of alternating loads was faster than the viscoelastic relaxation time of the DE material. This fact means that the actuation strain was not completely relaxed in each cycle, which strongly affected the deformation ability of the DEAs. 

The above results demonstrated that electromechanical coupling characteristics of the DEs when subjected step and alternating voltage presented a nonlinear and nonequilibrium process and energy was dissipated during the loading procedure. To account for these nonlinear behaviors of the DEs with an effective model was the main objective of this paper. 

## 3. Constitutive Modeling

The experimental results discussed above demonstrated that a DE system under voltage excitation exhibited complex, time-dependent, and nonlinear responses. To study the response characteristics of a DE membrane experiencing in-plane deformation, we focused on a widely used configuration in which a membrane of DE was sandwiched between two electrodes. In the reference state with no deformation, the initial length, width, and thickness of the DE membrane were $L_{1}$, $L_{2}$, and H, respectively. Subject to the in-plane mechanical forces $P_{1}$ and $P_{2}$, the DE membrane was in a pre-stretched state, where the length and width of the membrane changed to $L_{1pre}=L_{1}\lambda_{1pre}$ and $L_{2pre}=L_{2}\lambda_{2pre}$. We express the stretching ratio in the thickness direction as $\lambda_{3}=\lambda_{1}^{-1}\lambda_{2}^{-1}$ [26], meaning $H_{pre}=H/\lambda_{1pre}\lambda_{2pre}$. The two in-plane pre-stresses on the DE are defined as follows:

(1) $$\sigma_{1}=P_{1}/L_{2pre}H_{pre}=P_{1}\lambda_{1pre}/L_{2}H$$

(2) $$\sigma_{2}=P_{2}/L_{1pre}H_{pre}=P_{2}\lambda_{2pre}/L_{1}H$$

where $\sigma_{1}$ and $\sigma_{2}$ are the two in-plane actuation stresses of the DEs.

As we subjected a voltage U through the thickness of the membrane, a dipole phenomena was produced on the two electrodes. The Maxwell force was generated by the attraction of charges between the two electrodes. The membrane expanded in area and shrunk in thickness, and the system reached a new equilibrium state with dimensions $l_{1}=L_{1}\lambda_{1}$, $l_{2}=L_{2}\lambda_{2}$, and $h=H/\lambda_{1}\lambda_{2}$. Based on the assumption that the DE membrane acted as a parallel capacitor with compliant electrodes, the relationship between the charges Q and applied voltage U could be expressed as follows [28,29,30]:

(3) $$Q=U\frac{\varepsilon L_{1}L_{2}}{H}\lambda_{1}^{2}\lambda_{2}^{2}$$

where ε is the dielectric permittivity of the DE membrane. When the DE membrane was subjected to force and voltage, the variables in Equation (3) are $\lambda_{1}$, $\lambda_{2}$, and U, the variation in the charge is

(4) $$\delta Q=\frac{\varepsilon L_{1}L_{2}}{H}\lambda_{1}^{2}\lambda_{2}^{2}\delta U+\frac{\varepsilon L_{1}L_{2}}{H}U\left(2\lambda_{1}\lambda_{2}^{2}\delta \lambda_{1}+2\lambda_{2}\lambda_{1}^{2}\delta \lambda_{2}\right)$$

**Figure 5:**
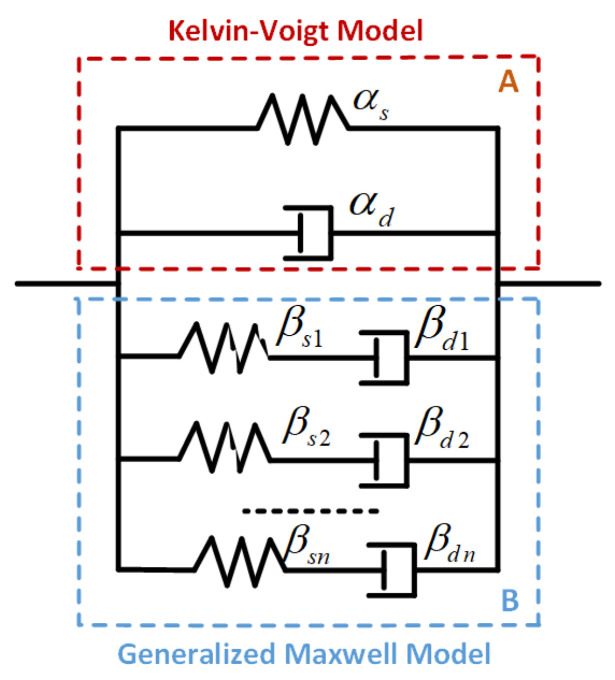
The Kelvin-Voigt-Generalized Maxwell (KV-GM) model.

In view of the related works, the Kelvin-Voigt model can better describe the initial creeping behavior [24], and the generalized Maxwell model is usually adopted to characterize the relaxation stage [25]. Therefore, in this paper, we parallelly connect both models and name it the Kelvin-Voigt-Generalized Maxwell model (KV-GM) as illustrated in Figure 5. In this model, we define $\lambda_{\alpha }$ and $\lambda_{\beta i}$ as the stretching factors in springs $\alpha_{s}$ and $\beta_{si}$, $\xi_{\alpha }$ and $\xi_{\beta i}$ as the stretching factors in dashpots $\alpha_{d}$ and $\beta_{di}$. By referring to the KV-GM model shown in Figure 5, the total stretching factor of the DE membrane was $\lambda =\lambda_{\alpha }=\xi_{\alpha }$. The corresponding net stretching factors of the two parallel parts were equal; thus, by adopting the well-established multiplication rule, we have $\lambda =\lambda_{\beta }\xi_{\beta }$ [31].

To describe the free energy density associated with the stretching of the DE, the Gent model [32] was adopted, which revealed the strain-stiffening performance of the DE. The strain energy of the DE was stored in the springs $\alpha_{s}$ and $\beta_{si}$, and thus the free energy density function could be expressed as follows:

(5) $$W=-\frac{\mu^{\alpha }J^{\alpha }}{2}\log\left(1-\frac{\lambda_{1}^{2}+\lambda_{2}^{2}+\lambda_{1}^{-2}\lambda_{2}^{-2}-3}{J^{\alpha }}\right)-\sum_{i=1}^{n}\frac{\mu_{i}^{\beta }J_{i}^{\beta }}{2}\log\left(1-\frac{\lambda_{1}^{2}\xi_{1i}^{-2}+\lambda_{2}^{2}\xi_{2i}^{-2}+\lambda_{1}^{-2}\lambda_{2}^{-2}\xi_{1i}^{2}\xi_{2i}^{2}-3}{J_{i}^{\beta }}\right)+\frac{D^{2}}{2e}$$

where $\mu_{\alpha }$ and $\mu_{\beta }$ are the shear moduli, and $J^{\alpha }$ and $J^{\beta }$ are the extension limits of the springs in parts A and B, respectively.

When the stretching of the DE membrane varied slightly in the two in-plane directions by $\delta \lambda_{1}$ and $\delta \lambda_{2}$, the tensile forces perform work that could be calculated as $P_{1}L_{1}\delta \lambda_{1}+P_{2}L_{2}\delta \lambda_{2}$. The corresponding increments in the charges of the two electrodes occurred with a small magnitude of δQ and the work done by the applied voltage was UδQ. During system actuation, the dashpot performed negative work and dissipated energy. The work done by the damping forces in part A is calculated as $\frac{1}{2}\eta_{\alpha }L_{1}^{2}\frac{d\xi_{\alpha 1}}{dt}\delta \xi_{\alpha 1}$ and $\frac{1}{2}\eta_{\alpha }L_{2}^{2}\frac{d\xi_{\alpha 2}}{dt}\delta \xi_{\alpha 2}$ [32], where is $\eta_{\alpha }$ the viscous damping coefficient of the dashpot $\alpha_{d}$, and $\xi_{\alpha 1}$ are $\xi_{\alpha 2}$ the stretching factors of dashpot $\alpha_{d}$ in the two in-plane directions. The work performed by the dashpot $\beta_{di}$ of in B in the two in-plane directions are $\sum_{i}\frac{1}{2}\eta_{\beta i}L_{1}^{2}\frac{d\xi_{\beta i1}}{dt}\delta \xi_{\beta i1}$ and $\sum_{i}\frac{1}{2}\eta_{\beta i}L_{2}^{2}\frac{d\xi_{\beta i2}}{dt}\delta \xi_{\beta i2}$, where $\eta_{\beta i}$ is the viscous damping coefficient of the dashpot $\beta_{di}$, $\xi_{\beta i1}$ and $\xi_{\beta i2}$ are the stretching factors of the dashpot $\beta_{d}$ in the two in-plane directions. During the actuation process, the work performed by the inertial force in the two in-plane directions was calculated as $\frac{\rho L_{1}^{2}L_{2}H}{3}\frac{d^{2}\lambda_{1}}{dt^{2}}\delta \lambda_{1}$ and $\frac{\rho L_{1}L_{2}^{2}H}{3}\frac{d^{2}\lambda_{2}}{dt^{2}}\delta \lambda_{2}$ [33], where ρ is the density of VHB4910 elastomer. Thermodynamic principles state that the arbitrary variation of a viscoelastic DE system should be equal to the work performed by the applied voltage, tensile force, damping force, and inertia force. The function of the free energy density is defined in (6).

(6) $$\begin{array}{l} L_{1}L_{2}H\delta W=\phi \delta Q+\sigma_{1}L_{1}^{2}H\delta \lambda_{1}+\sigma_{2}L_{2}^{2}H\delta \lambda_{2}-\left(\frac{1}{2}\eta_{\alpha }L_{1}^{2}\frac{d\xi_{\alpha 1}}{dt}\delta \xi_{\alpha 1}+\frac{1}{2}\eta_{\alpha }L_{2}^{2}\frac{d\xi_{\alpha 2}}{dt}\delta \xi_{\alpha 2}\right)\\ -\left(\frac{\rho L_{1}^{2}L_{2}H}{3}\frac{d^{2}\lambda_{1}}{dt^{2}}\delta \lambda_{1}+\frac{\rho L_{1}L_{2}^{2}H}{3}\frac{d^{2}\lambda_{2}}{dt^{2}}\delta \lambda_{2}\right)-\left(\sum_{i=1}^{n}\frac{1}{2}\eta_{\beta i}L_{1}^{2}\frac{d\xi_{\beta 1i}}{dt}\delta \xi_{\beta 1i}+\sum_{i=1}^{n}\frac{1}{2}\eta_{\beta i}L_{1}^{2}\frac{d\xi_{\beta 2i}}{dt}\delta \xi_{\beta 2i}\right) \end{array}$$

In the following discussion, we consider a special case in which a DE membrane is under equal biaxial stress, that is, $\sigma_{1}=\sigma_{2}=\sigma$ and $L_{1}=L_{2}=L$. Assuming that the stretching in the dashpot $\alpha_{d}$ is consistent with the total stretching of the DE, we could derive $\lambda_{1}=\lambda_{2}=\lambda$, $\xi_{\alpha 1}=\xi_{\alpha 2}=\xi_{\alpha }=\lambda$, and $\xi_{\beta i1}=\xi_{\beta i2}=\xi$. Therefore, (5) and (6) could be rewritten as

(7) $$W=-\frac{\mu^{\alpha }J^{\alpha }}{2}\log\left(1-\frac{2\lambda^{2}+\lambda^{-4}-3}{J^{\alpha }}\right)-\sum_{i}\frac{\mu_{i}^{\beta }J_{i}^{\beta }}{2}\log\left(1-\frac{2\lambda^{2}\xi_{\beta i}^{-2}+\lambda^{-4}\xi_{\beta i}^{4}-3}{J^{\beta }}\right)+\frac{D^{2}}{2e}$$

(8) $$L^{2}H\delta W=\phi \delta Q+2\sigma L^{2}H\delta \lambda -\eta_{\alpha }L^{2}\frac{d\lambda }{dt}\delta \lambda -\sum_{i}\eta_{\beta i}L^{2}\frac{d\xi_{\beta i}}{dt}\delta \xi_{\beta i}-\frac{\rho L^{3}H}{3}\frac{d^{2}\lambda }{dt^{2}}\delta \lambda$$

The effects of the inertial force on the DE system could be ignored under step-voltage excitation. Based on the standard calculus of variation in (7), we found that:

(9) $$\frac{\partial W}{\partial \lambda }=\frac{2\mu_{\alpha }\left(\lambda -\lambda^{5}\right)}{1-\left(2\lambda^{2}+\lambda^{-4}-3\right)/J^{\alpha }}+\sum_{i}\frac{2\mu_{\beta i}\left(\lambda \xi_{i}^{-2}-\lambda^{-5}\xi_{i}^{-2}\right)}{1-\left(2\lambda^{2}\xi_{i}^{-2}+\lambda^{-4}\xi_{i}^{-2}-3\right)/J_{i}^{\beta }}\;(a)\;\frac{\partial W}{\partial \xi_{i}}=-\frac{2\mu_{\beta i}\left(\lambda^{2}\xi_{i}^{-3}-\lambda^{-4}\xi_{i}^{3}\right)}{1-\left(2\lambda^{2}\xi_{i}^{2}+\lambda^{-4}\xi_{i}^{4}-3\right)/J_{i}^{\beta }}\;(b)\;\frac{\partial W}{\partial D}=\frac{D}{\varepsilon }\;(c)$$

Based on (4), replacing the charge Q with the electrical displacement D yielded

(10) $$\frac{\partial W}{\partial \lambda }=\frac{2\phi D}{H}\lambda +2\sigma -\frac{\eta_{\alpha }}{H}\frac{d\lambda }{dt}\;(a)\;\frac{\partial W}{\partial \xi }=\sum_{i}^{}-\frac{\eta_{\beta i}}{H}\frac{d\xi }{dt}\;(b)\;\frac{\partial W}{\partial D}=\frac{\phi }{H}\lambda^{2}\;(c)$$

Considering the work performed by the inertia force, for a DE system under the excitation of an alternating load, (8) could be rewritten as

(11) $$\frac{\delta W}{\delta \lambda }=\varepsilon {\left(\frac{\phi }{h}\right)}^{2}\lambda^{3}-\frac{\eta_{\alpha }}{H}\frac{d\lambda }{dt}-\frac{2\rho L^{2}}{3}\frac{d^{2}\lambda }{dt}+2\sigma$$

By combining (9) and (10) and eliminating the electrical displacement D, the governing equations could be expressed as follows:

(12) $$\frac{d\lambda }{dt}=\frac{2H}{\eta_{\alpha }}\left(\frac{\varepsilon \phi^{2}}{H^{2}}\lambda^{3}+\sigma -\frac{\mu_{\alpha }\left(\lambda -\lambda^{-5}\right)}{1-\left(2\lambda^{2}+\lambda^{-4}-3\right)/J^{\alpha }}-\sum_{i}^{}\frac{\mu_{\beta i}\left(\lambda \xi_{i}{}^{-2}-\lambda^{-5}\xi_{i}{}^{4}\right)}{1-\left(2\lambda^{2}\xi_{i}{}^{-2}+\lambda^{-4}\xi_{i}{}^{4}-3\right)/J^{\beta }}\right)$$

(13) $$\frac{d\xi_{i}}{dt}=\frac{2H\mu_{\beta i}}{\eta_{\beta i}}\frac{\lambda^{2}\xi_{i}{}^{-3}-\lambda^{-4}\xi_{i}{}^{3}}{1-\left(2\lambda^{2}\xi_{i}{}^{-2}+\lambda^{-4}\xi_{i}{}^{4}-3\right)/J^{\beta }}$$

By substituting (11) into (9), we obtained the following dynamic equations for the system:

(14) $$\frac{2\rho L^{2}}{3}\frac{d^{2}\lambda }{dt^{2}}+\frac{\eta_{\alpha }}{H}\frac{d\lambda }{dt}-\varepsilon {\left(\frac{\phi }{H}\right)}^{2}\lambda^{3}+\frac{2\mu_{\alpha }\left(\lambda -\lambda^{-5}\right)}{1-\left(2\lambda^{2}+\lambda^{-4}-3\right)/J^{\alpha }}+\sum_{i}\frac{2\mu_{\beta }\left(\lambda \xi_{I}^{-2}-\lambda^{-5}\xi_{I}^{4}\right)}{1-\left(2\lambda^{2}\xi^{-2}+\lambda^{-4}\xi_{I}^{4}-3\right)/J^{\beta }}-2\sigma =0$$

(15) $$\frac{d\xi_{i}}{dt}=\frac{2H\mu_{\beta I}}{\eta_{\beta I}}\frac{\lambda^{2}\xi_{I}^{-3}-\lambda^{-4}\xi_{I}^{3}}{1-\left(2\lambda^{2}\xi^{-2}+\lambda^{-4}\xi_{I}^{4}-3\right)/J^{\beta }}$$

**Figure 6:**
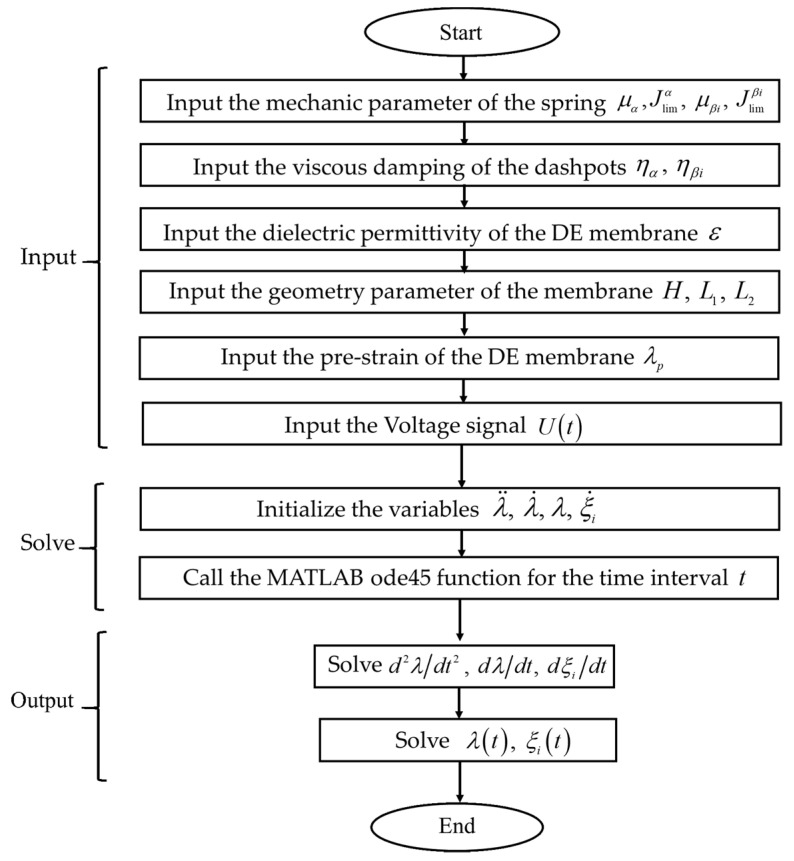
Algorithm for solving the governing equations of the DE.

An algorithm for solving the DE equations is shown in Figure 6. The length of the DE membrane was, $L_{1}=L_{2}=L=0.01\;m$ and its thickness was H=0.001 m. The density of the VHB4910 elastomer was $\rho =960\;\mathrm{kg}/m^{3}$ [33]. The permittivity of DE was $\varepsilon =4.11*10^{-11}\;F/m$ [26]. In the relaxed state, we applied the initial conditions of λ(0)=3, dλ(0)/dt=0, $d^{2}\lambda \left(0\right)/dt^{2}=0$, and $d\xi_{i}\left(0\right)/dt=3$. The Dormand-Prince method from the Runge-Kutta ODE family of solvers family was adopted in this study to solve the ordinary differential mathematical model. All numerical calculations were conducted using the ode45 function in MATLAB.

## 4. Parameter Identification

**Figure 7:**
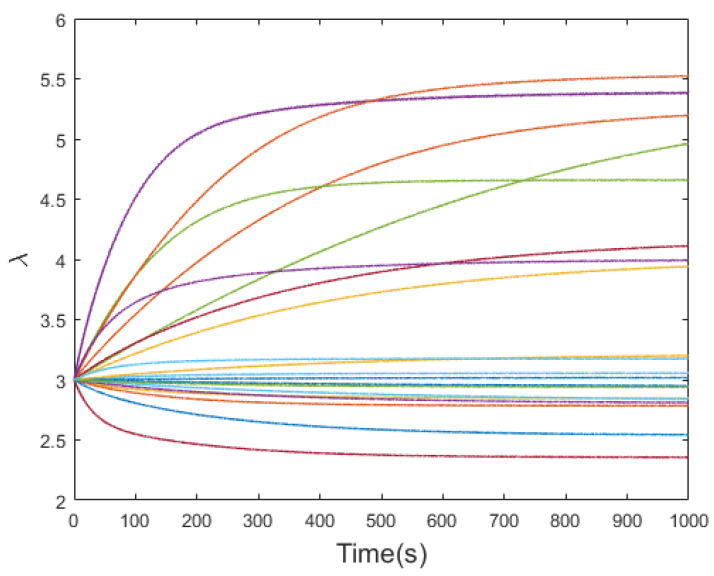
First-order KV-GM rheological model system responses for 20 random Monte-Carlo samples.

To describe the responses of the DE membrane quantitatively under the excitation of step voltage and alternating voltage, based on a prediction model, we first analyzed the influence of the model parameters on the system responses. To simplify the analysis process, the model used in our simulation testing was a single Maxwell unit KV-GM model, and a Monte Carlo static simulation was used as an auxiliary method for parameter identification. The Monte Carlo method is a numerical method based on probability and statistical theory. Random numbers are used to solve many types of computational problems. The unknown parameters in the model were randomly sampled several times. The sampling centers of the parameters given in [15] were used as the basic values. The sampling ranges were adjusted appropriately according to the load applied by the system. Figure 7 presents the simulation results of the first-order KV-GM rheological constitutive model responses for 20 random samples. The results demonstrated that the effective range of the parameters could be determined using random sampling. Additionally, one could see that the failure ranges of the parameters caused the system responses to fall below the initial values. In the following model parameter identification process, a multi-order rheological model combined with Monte Carlo multiple sampling was used to obtain an optimal parameter combination to improve the descriptive ability of the model.

### 4.1. Step Voltage Excitation

**Figure 8:**
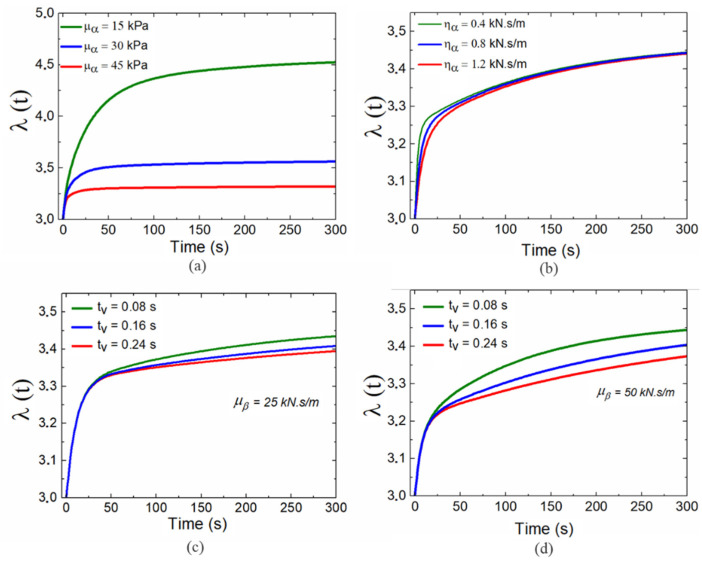
The influence of model parameters in step response: (**a**) the response of the model under different shear modulus of the spring $\alpha_{s}$, (**b**) the response of the model under different viscous damping of the dashpot $\alpha_{d}$, (**c**) the response of the model under different relaxation time with $\mu_{\beta }=25\mathrm{kN}.s/m$, (**d**) the response of the model under different relaxation time with $\mu_{\beta }=50\mathrm{kN}.s/m$.

**Figure 9:**
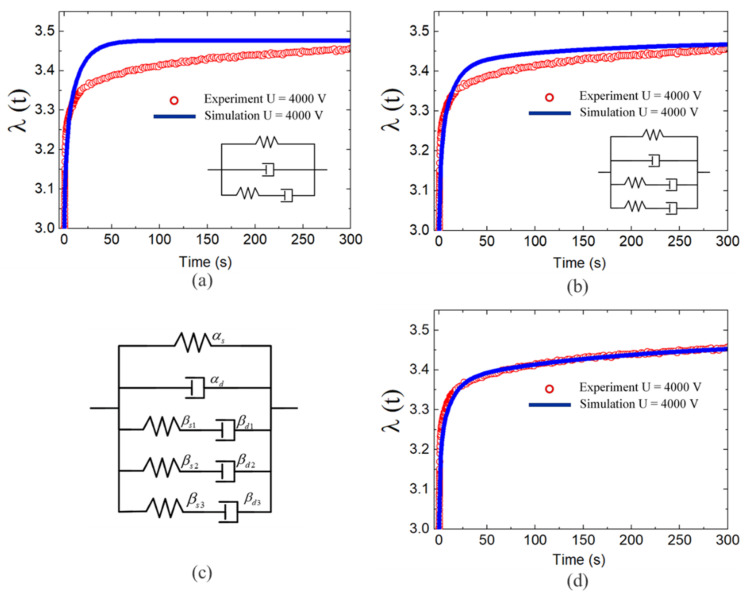
Step quantitative analysis of experimental and simulation results for DE membranes with different numbers of Maxwell units: (**a**) one-unit fitting, (**b**) two-unit fitting, (**c**) effective rheological model, and (**d**) effective three-unit fitting.

The responses of the system under a step voltage could be modeled using (12) and (13) by ignoring the influence of the inertial force. First, we attempted to analyze the influence of the model parameters on the response process of the DE system. By considering different stretch limit parameters, it could be concluded that stretch limits do not have a significant effect on the model responses. The reason for this phenomenon may be that the material experiences deformation smaller than the stretch limit [25]. To optimize the model parameters, we set up a prediction model to investigate the response characteristics of models with different material parameters. The optimal parameters were then determined by quantitatively comparing the experimental results to describe the step responses of the system. During this prediction process, the voltage U = 4000 V was used as the step excitation load, and different material parameters were considered to ensure that the system responses did not generate distortion phenomena. To simplify the analysis process, we considered that the step responses of the DE membrane could be divided into three stages: the first stage of initial rapid deformation, the second stage of slow creeping and relaxation, and the third stage of a steady-state. The simulation results demonstrated that the shear modulus $\mu_{\alpha }$ of the spring $\alpha_{s}$ in part A of the rheological model mainly affected the third stage of the system, as shown in Figure 8a. It was remarkable that the steady-state exhibited greater deformation when the shear modulus was small. This greater deformation occurred because a decrease in the shear modulus implied that the material was softer. Therefore, under the same external stress, the softer material experienced a larger deformation. Subsequently, by considering the influence of the viscous damping $\eta_{\alpha }$ of the dashpot $\alpha_{d}$ in part A, as shown in Figure 8b, we could determine that different values for $\eta_{\alpha }$ mainly affected the rate of increase in the first stage of the initial rapid deformation. An increase in viscous damping $\eta_{\alpha }$ caused the creep deformation to drift more slowly. This phenomenon was attributed to the fact that greater viscous damping leads to a large resistance to limited deformation, thereby increasing the creep time. The influence of part B of the rheological model could be examined with different relaxation times $t_{v}=\eta_{b}/\mu_{b}$, as shown in Figure 8c. According to the definition of relaxation time, it is trivial to conclude that an increase in relaxation time would extend the relaxation process in the second stage of the model response, meaning that the system would require more time to reach the steady-state. Figure 8c,d present the comparative model responses with different shear modulus for part B. The shear modulus directly affected the initial deformation in the second stage of the response process. This phenomenon was easy to understand because for a given relaxation time, increasing the shear modulus of the Maxwell element will limit the deformation of the DE material. According to the model parameter analysis above, the shear modulus in part A should be set at $\mu_{\alpha }=35\;\mathrm{kPa}$ to ensure steady deformation of the system in the final stage. Additionally, the viscous damping in part A $\eta_{\alpha }=0.2\;\mathrm{kN}.s/m$ should generate a suitable rate of increase in the model response in the initial creep stage. Based on the comparative results in Figure 9c,d, the shear modulus of the Maxwell element was $\mu_{\beta }=15\;\mathrm{kPa}$. This calculation was an important step in determining the initial deformation of the system during the relaxation stage. 

Figure 9a,b,d present the prediction results for the rheological model with different orders of Maxwell units. Figure 9a presents the fitting results for a rheological model with a single unit and a single-order relaxation time of $t_{v1}=0.008\;s$. It could be seen that there was a notable difference between the simulation and experimental results. Figure 9b presents the fitting results for the two Maxwell units with $t_{v1}=0.008\;s$ and $t_{v2}=0.067\;s$. It could be clearly seen that increasing the number of Maxwell units reduced the error in the fitting results. The three Maxwell units in the rheological model in Figure 9c accurately describe the response of the DE membrane under a complex step load. The parameters used for this three-order rheological model were $t_{v1}=0.008\;s$, $t_{v2}=0.067\;s$, and $t_{v3}=0.167\;s$. Figure 9d shows that the model predictions and experimental results were in good agreement throughout the step-response process.

### 4.2. Alternating Voltage Excitation

**Figure 10:**
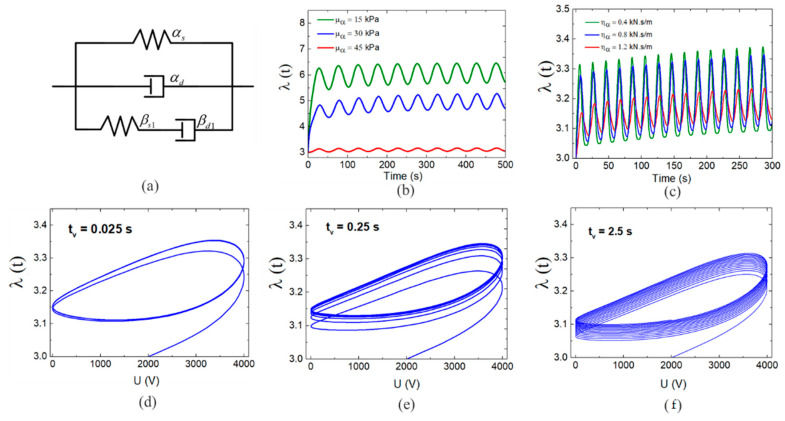
Influence of model parameters on alternating voltage responses: (**a**) one Maxwell unit rheological model, (**b**) the response of the model under different shear modulus of the spring $\alpha_{s}$, (**c**) the response of the model under different viscous damping of the dashpot $\alpha_{d}$, (**d**–**f**) the hysteresis loop of the model response under different relaxation time.

We now considered excitation by an alternating voltage $U=U_{0}\sin2\pi ft$ with $U_{0}=2\mathrm{kV}\left(V\right)$ and f=0.05 Hz. By considering the influence of the inertial force, we obtained a dynamic model for a DE system, as defined in (14) and (15). Figure 10a presents the system responses with different shear modulus for the spring $\alpha_{s}$ in part A of the rheological model. The results demonstrated that a smaller shear modulus leads to a smaller deformation in the model response. This phenomenon could be interpreted as follows. A lower elastic model indicates that a material is softer, and a softer material experiences a larger deformation under a given stress. Figure 10b presents the effects of various viscous damping coefficients for the dashpot $\alpha_{d}$ in part A. It was clear that an increase in viscous damping could lead to an attenuation of the vibration amplitude. Larger viscous damping restricted the vibration of the DE membrane. The membrane would not deform because of this restriction, and it would return to the equilibrium position.

The influence of a single spring-dashpot unit in part B could be obtained by analyzing different values of relaxation time, $t_{v}=\eta_{b}/\mu_{b}$ as shown in Figure 10d,e,g. With increasing relaxation time, the system response exhibited clear creep effects. In a system with very little creep, equilibrium was achieved very quickly, as shown in Figure 10d. When the creep process requires a long time, several cycles were required to reach equilibrium, as shown in Figure 10g. According to the analysis above, the shear modulus and viscous damping of part A were determined first to obtain a stable positional deformation and vibration amplitude for the system. Next, we set the relaxation time to adjust the creep and relaxation processes of the model response. According to the experimental results, the model parameters for Part A should be $\mu_{\alpha }=35\;\mathrm{kPa}$ and $\eta_{\alpha }=0.15\;\mathrm{kN}.s/m$.

**Figure 11:**
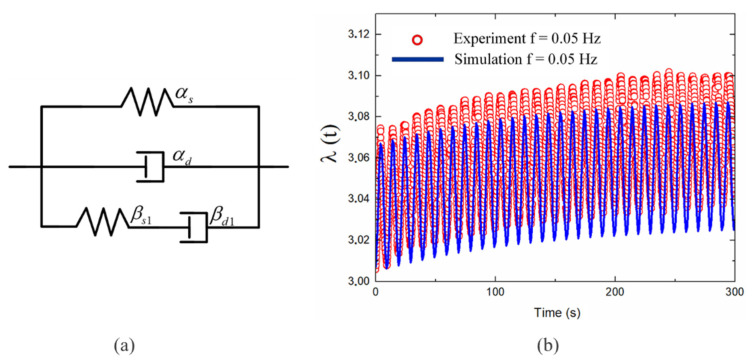
Alternating voltage comparative analysis between experimental results and simulation results for a DE membrane with a single Maxwell unit: (**a**) one Maxwell units, (**b**) one-Maxwell-unit fitting results.

The results in Figure 11b indicated that the amplitudes of the model responses were similar to those of the experimental results in the first several periods. If the shear modulus of the Maxwell unit remained invariant, the influence of different relaxation times on the model responses could be obtained by varying the viscous damping. As shown in Figure 10d,e,g, increasing the viscous damping could lead to the attenuation of the vibration amplitude. Therefore, to guarantee the amplitude of the model responses, we maintained $\eta_{\beta }=6\;\mathrm{kN}.s/m$ and varied the shear modulus of the spring-dashpot unit to adjust the relaxation time.

**Figure 12:**
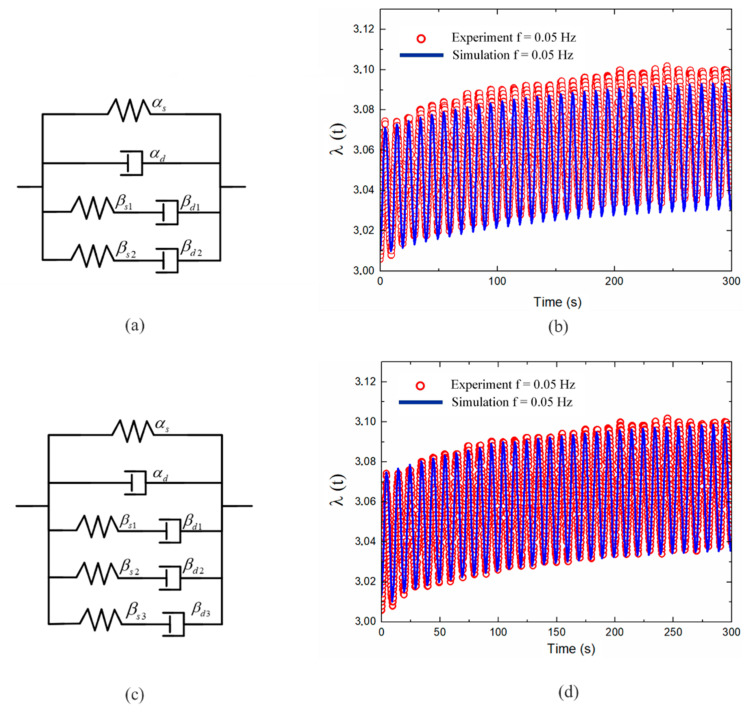
Alternating voltage comparative analysis between experimental results and simulation results for DE membranes with different numbers of Maxwell units: (**a**) two Maxwell units, (**b**) two-Maxwell-unit fitting results, (**c**) three Maxwell units, (**d**) three-Maxwell-unit fitting results.

A simulation experiment was conducted with a single Maxwell unit, as shown in Figure 11b, and a second-order Maxwell unit, as shown in Figure 12b, to determine the optimal relaxation time for part B. The results revealed that $t_{v1}=0.092\;s$ for a single Maxwell unit and, $t_{v1}=0.092\;s$ and $t_{v2}=0.13\;s$ for two Maxwell units. Overall, the model responses closely fitted the experimental results for the first three cycles of the significant creep process, but errors occurred in the relaxation stage. However, the error decreased with an increase in the number of Maxwell units. To reduce the error, a three-order Maxwell model was applied with, $t_{v1}=0.092\;s$, $t_{v2}=0.13\;s$, and $t_{v3}=0.6\;s$. Figure 12d shows good agreement between the model predictions and the experimental results throughout the response process. 

## 5. Discussion

To further quantify the performance of the KV-GM model, the maximum prediction error $e_{m}$ and the root-mean-square error $e_{rms}$ mean are defined as follows:

(16) $$e_{m}=\max\frac{\left|\lambda_{s}-\lambda_{e}\right|}{\max(\lambda_{e})-\min(\lambda_{e})}$$

(17) $$e_{rms}=\frac{\sqrt{\frac{1}{N}\sum_{i=1}^{N}{\left(\lambda_{s}(i)-\lambda_{e}(i)\right)}^{2}}}{\max(\lambda_{e})-\min(\lambda_{e})}$$

where $\lambda_{s}$ and $\lambda_{e}$ present predicted results and experimental data, respectively, and N is the number of measurements. 

**Figure 13:**
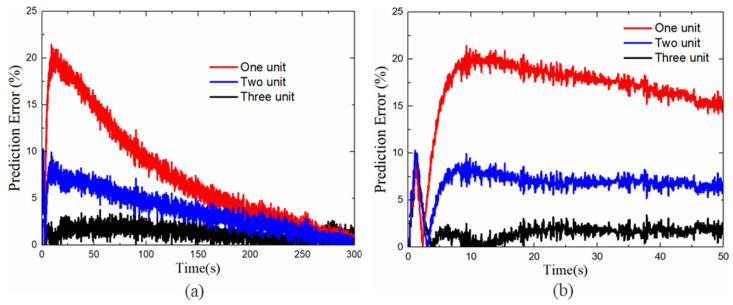
Prediction errors under step voltage excitation for DE membranes with different numbers of Maxwell units: (**a**) the prediction errors, (**b**) The initial 50 s of prediction error.

Figure 13a shows the prediction errors of different numbers of Maxwell units with step voltage excitation. The initial 50 s prediction error is shown in Figure 13b. It can be found that the overshot error occurs in the first 5 s, this phenomenon can be explained as prior to entering the long time relaxation process, the response of displacement mutation and creep stage is relatively complex, which increases the difficulty of the model prediction. On the other hand, the prediction at this stage is mainly determined by the two fixed parameters of Part A of the rheological model as described in Section 4. As shown in Figure 13b, when the Maxwell element of the model increases, the overshoot values are 8.841%, 10.26%, 8.931%, respectively. These results show that the change of the Maxwell unit basically does not affect the prediction ability in the initial response state. Therefore, to evaluate the effectiveness of different Maxwell element models, we only consider the error values after the overshoot process. The maximum prediction error of the step voltage excitation and root-mean-square error of the alternating voltage excitation are listed in Table 1. We found that the increase of Maxwell unit numbers can reduce the prediction error. It should be noted that a further increase in the numbers of the Maxwell unit (more than 3) causes a further increase in the accuracy of the model, but it will increase the cost of computing. Without loss of generality, in this work, we used three Maxwell units as a research object.

**Figure 14:**
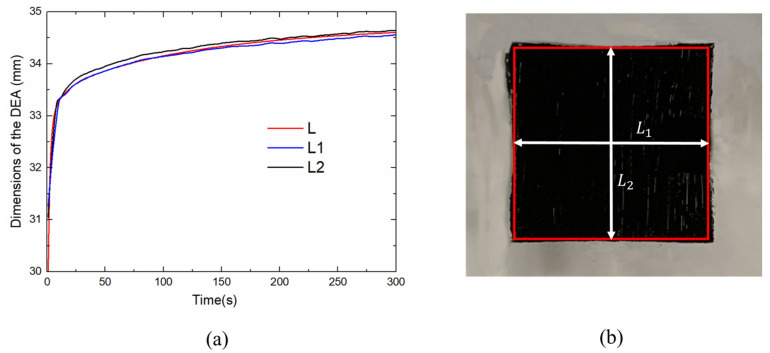
Experimental results of the DEA under two measurement methods: (**a**) The dynamic dimensions of two in-plane directions under U=4kV voltage excitation, (**b**) Geometrical shape.

The side lengths of the rectangle DEA are shown in Figure 14a, where L is the side length of the square calculated by our theoretical model, $L_{1}$ and $L_{2}$ are the length and width of the rectangle measured by two laser displacement sensors. The discrepancy of $L_{1}$ and $L_{2}$ indicates that the actuator deforms slightly differently in two in-plane directions. These discrepancies mainly come from the hand-made pre-stretching process, which leads to the tensile force of DES film in the two directions is not exactly the same before actuation. Figure 14b presents an actuation area by CCD camera, the rectangle in red is the shape of $L_{1}$ and $L_{2}$. It is found that the edges of the rectangle are no longer straight, which results in a distortion of the rectangle. For DEs membrane, a frame is used to hold the membrane after pre-stretching, the passive region on the film (flexible electrode uncoated) is limited in area and then results in concentrated stress distribution. During the actuation, it causes uneven tensile force and varies the boundary conditions of the actuator, and results in excessive deformation at the corners.

To further verify the validity of the proposed model and the shape of the DEA in the actuation process, we consider the area deformation of the DEA under a different magnitude of step voltages. The measurement area $S_{measure}$ and estimation area $S_{estimate}$ and true area $S_{camera}$ are defined, respectively, as follows:

(18) $$S_{measure}=L_{1\_measure}\times L_{2\_measure}$$

(19) $$S_{estimate}=L_{1\_estimate}\times L_{2\_estimate}$$

where $L_{1\_measure}$ and $L_{2\_measure}$ is the length of the actuator detected by the displacement sensor in the two in-plane directions, $L_{1\_estimate}$ and $L_{2\_estimate}$ is the length of the actuator calculated by the proposed model in the two in-plane directions.

To verify the validity of the proposed model, we can define the prediction error of the actuator area as follows:

(20) $$Error_{m\_e}=\frac{S_{measure}-S_{estimate}}{S_{measure}}$$

Then, to further verify that the actuator whether can maintain a precise rectangular shape after the pre-stretching and excitation process, we defined $Error_{c\_m}$ error expressed as:

(21) $$Error_{c\_m}=\frac{S_{camera}-S_{measure}}{S_{measure}}$$

where $S_{camera}$ is the area recorded by the CCD external trigger camera.

**Figure 15:**
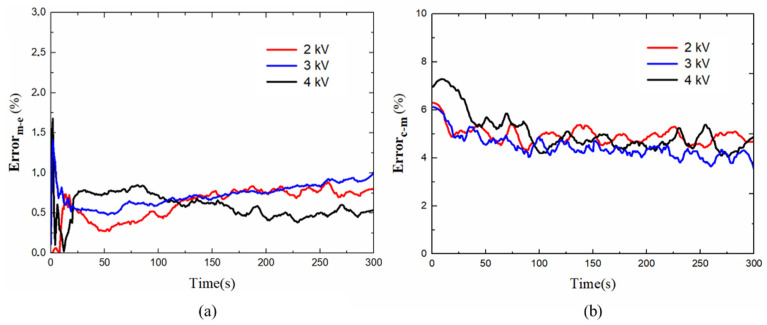
The area errors of the DEA under different excitation voltages: (**a**) $Error_{m\_e}$ and (**b**) $Error_{c\_m}$.

The prediction area errors $Error_{m\_e}$ of the actuator area under different step voltages are shown in Figure 15a and the $Error_{c\_m}$ of the planar DEA as shown in Figure 15b. Table 2 shows the maximum value of both errors. In Figure 15a,b, the maximum error mainly occurs in a few seconds at the initial stage. For the displacement mutation and creeping at the very beginning, the actuator can not be able to relax completely in time, which leads to an increase in the prediction area error. When the voltage increases, the velocity of the deformation grows and the nonlinear viscosity is more significant, the response of the actuator becomes more complex, thus making the prediction of the model more difficult and leads to the error in the initial stage increases with the excitation voltage. After entering the long relaxation phase, the actuator has enough time to relax completely, and the error is smaller. The maximum area errors were 1.674% and 7.485%, respectively, indicating that the proposed model agrees well with experimental measurements and the actuator shape can basically maintain a precise rectangular shape during pre-stretching and high voltage excitation.

## 6. Conclusions

The complex electromechanical coupling response of the DEs is discussed in this study. First, different voltage signals are applied to planar DEA to understand strong nonlinear phenomena coupled with the complex time and frequency-dependent viscoelasticity of DEs. Then, a generalized rheological model is proposed based on the principle of virtual work, and non-equilibrium thermodynamics presents a high-precision prediction ability with three relaxation times. Furthermore, the model parameter identification method proposed in this paper can identify a set of optimal parameters by combining model parameter analysis and the Monte Carlo statistical simulation method. The final results demonstrate that the generalized rheological model can accurately predict the responses of DE materials under high step voltages with a maximum prediction error of 3.762% and complex alternating voltages with a maximum root-mean-square prediction error of 9.03%. To further verify the validity of the proposed model and the shape of the actuator during the actuation process, under different step voltages excitation, the maximum prediction area error reached 1.674% and the maximum error obtained by the two measurements methods is 7.485%. These results have strongly contributed to the high-precision control of DE material systems and enhanced the field of flexible robotics.

## Figures and Tables

**Table 1:** Prediction errors at different numbers of Maxwell unit.

Unit Numbers	Step Voltage, $e_{m}(\%)$	Alternating Voltage, $e_{rms}(\%)$
One unit	21.42	15.796
Two unit	9.936	11.553
Three unit	3.762	9.03

**Table 2:** Maximum errors of the effective three units modeled at different excitation voltages.

Voltage	$Error_{m\_e}$, (%)	$Error_{c\_m}$, (%)
2 kV	1.113	6.149
3 kV	1.315	6.349
4 kV	1.674	7.485

## Data Availability

https://doi.org/10.6084/m9.figshare.14173697, accessed on 28 June 2021.
